# Supplementary material for: Van Krevelen diagrams based on machine learning visualize feedstock-product relationships in thermal conversion processes
Source: Commun Chem. 2023 Dec 13;6:273. doi: 10.1038/s42004-023-01077-z (PMC10716171; doi:10.1038/s42004-023-01077-z)
Supplement: Supplementary file 2 — Supplementary information [file 42004_2023_1077_MOESM2_ESM.pdf]

# Supplementary information

## Van Krevelen diagrams based on machine learning visualize feedstock-product relationships in thermal conversion processes

Shule Wang[1,2], Yiying Wang[3], Ziyi Shi[4], Kang Sun[1,2], Yuming Wen[3]*, Lukasz Niedzwiecki[5,6], Ruming Pan[7,8], Yongdong Xu[9], Ilman Nuran Zaini[4], Katarzyna Jagodzińska[4], Christian Aragon-Briceno[10], Chuchu Tang[11], Thossaporn Onsree[12], Nakorn Tippayawong[13], Halina Pawlak-Kruczek[5], Pär Göran Jönsson[4], Weihong Yang[4], Jianchun Jiang[1,2]*, Sibudjing Kawi[3]*, Chi-Hwa Wang[3]*

*[1] Jiangsu Province Key Laboratory of Biomass Energy and Materials, National Engineering Laboratory for Biomass Chemical Utilization, Institute of Chemical Industry of Forest Products, Chinese Academy of Forestry (CAF), Nanjing, 210042, China.*

*[2] Jiangsu Co-Innovation Center for Efficient Processing and Utilization of Forest Resources, College of Chemical Engineering, Nanjing Forestry University, Longpan Road 159, Nanjing 210037, China.*

*[3] Department of Chemical and Biomolecular Engineering, National University of Singapore, 4 Engineering Drive 4, E5 #02-09, Singapore 117585.*

*[4] Department of Materials Science and Engineering, KTH Royal Institute of Technology, SE-100 44, Stockholm, Sweden.*

*[5] Department of Energy Conversion Engineering, Wroclaw University of Science and Technology, 27 wybrzeże Stanisława Wyspiańskiego st.*

*50-370 Wroclaw, Poland.*

*[6] Energy Research Centre, Centre for Energy and Environmental Technologies, VŠB-Technical University of Ostrava, 708 00, Ostrava – Poruba, Czech Republic*

*[7] School of Energy Science and Engineering, Harbin Institute of Technology, Harbin, 150001, China.*

*[8] Institut de Mécanique des Fluides de Toulouse (IMFT) - Université de Toulouse, CNRS-INPT-UPS, 31400 Toulouse, France.*

*[9] Laboratory of Environment-Enhancing Energy (E2E), Key Laboratory of Agricultural Engineering in Structure and Environment of Ministry of Agriculture and Rural Affairs, China Agricultural University, Beijing, 100083, China.*

*[10] Department of Industry and Energy, CIRCE-Research Centre for Energy Resources and Consumption, 50018, Zaragoza, Spain.*

*[11] Faculty of Creative Arts, University of Malaya, 50603, Kuala Lumpur, Malaysia.*

*[12] Department of Chemical Engineering, University of South Carolina, 301 Main St, Columbia, SC 29208, USA.*

*[13] Department of Mechanical Engineering, Chiang Mai University, 239 Huay Kaew Rd., Muang District, Chiang Mai, Thailand 50200*

These authors contributed equally: Shule Wang, Yiying Wang, Ziyi Shi, Kang Sun.

***Email:** Yuming Wen: yuming@nus.edu.sg; Jianchun Jiang: jiangjc@caf.ac.cn; Sibudjing Kawi: chekawis@nus.edu.sg; Chi-Hwa Wang: chewch@nus.edu.sg

## Supplementary Note 1

### Feedstock composed of multiple types of raw materials

Most of real biodegradable waste and biomass are a mixture feedstock. Collected biodegradable waste in real situation normally contains different types of raw materials such as sludge, sawdust, algae, etc. Hence, compared to the thermal treatments of a pure substance, the application of thermal treatment techniques is more complicated. This will also be reflected by the van Krevelen diagrams created in this study. The hydrothermal carbonization (HTC) diagrams are constructed by machine learning (ML) based on a training data consist of biodegradable waste. We use HTC diagrams as an example to make a discussion here.

The dehydration and decarboxylation are the main reactions during an HTC[1]. Taking this into consideration, one would expect that using data from extensive suits of experiments would result in showing some kind of a pattern following the pathway on the yielded van Krevelen diagrams, in which locations of products on Van Krevelen diagram would exhibit some sort of diagonal pattern, following certain angle between decarboxylation and dehydration. However, this is clearly not the case as shown in Supplementary Figure 1. The HTC process increases in complexity when mechanisms for feedstocks other than lignocellulosic biomass, such as food waste[2] or sewage sludge[3]. Mixing different types of feedstocks, adds further to the complexity of the process and carbonization pathways[4].

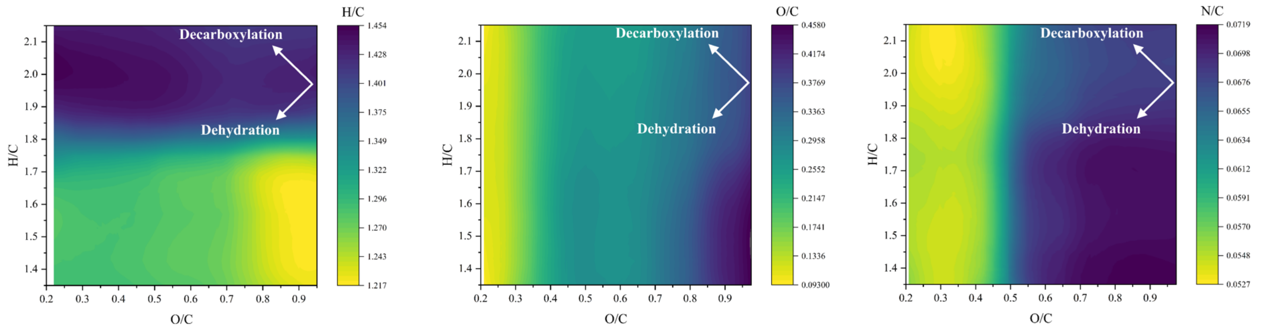


**Supplementary Figure 1. HTC diagrams of the H/C, O/C, and N/C of hydrochar.** The reaction directions of decarboxylation and dehydration are plotted in the diagrams.

## Supplementary Note 2

### Catalytic effect of ash on thermal conversion

Ash in feedstock represents the inorganic residue persisting post-combustion. Comprising elements like calcium, potassium, sodium, magnesium, silicon, phosphorus, sulfur, and chlorine, ash's influence on feedstock's thermal decomposition varies based on its composition and concentration. For example, biomass ash can either catalyze or inhibit specific reactions, potentially acting as a catalyst poison. When a catalyst is introduced to the thermal process, ash can lead to its deactivation through mechanisms such as coking, sintering, and poisoning. Moreover, ash can modify the quality and yield of thermal process products, including bio-oil, gas, and char derived from pyrolysis. Consequently, ash is a pivotal parameter in refining thermal conversion processes and associated catalysts.

In pyrolysis, ash profoundly impacts both product distribution and quality. Ash presence escalates non-condensable gas formation while diminishing yields of organic and aqueous liquid phases. This phenomenon can be traced back to the catalytic actions of ash constituents, notably alkali and alkaline earth metals, which can foster decarboxylation, dehydration, and cracking reactions[5].

The catalytic influence of ash in gasification accelerates the gasification reaction rate and carbon-containing material conversion by integrating biomass ash or other alkali metal compounds as catalysts. Rich in alkali metals like potassium and sodium, biomass ash can reduce activation energy and enhance the gasification process's reactivity. The catalytic impact of biomass ash is contingent upon various factors, including the biomass ash addition ratio, alkali metals' chemical forms in the ash, the fuel's mineral content, and the gasification temperature and environment. The underlying catalytic mechanism involves the creation of a molten or semi-molten alkali metal compound layer on the fuel char's surface, streamlining the reactant and product transfer between gas and solid phases. This catalytic influence can augment

gasification's efficiency and selectivity, potentially decreasing the operational temperature and capital expenditure[6].

While ash's catalytic effect in other thermal conversion processes isn't exhaustively discussed here due to length constraints, its significance in thermal conversion is undeniable. However, this study predominantly centers on the H/C and O/C ratios of the feedstock. Delving into ash's effects will be a subsequent focus in our machine learning research. Supplementary Figure 2 presents the range of the ash content in each database.

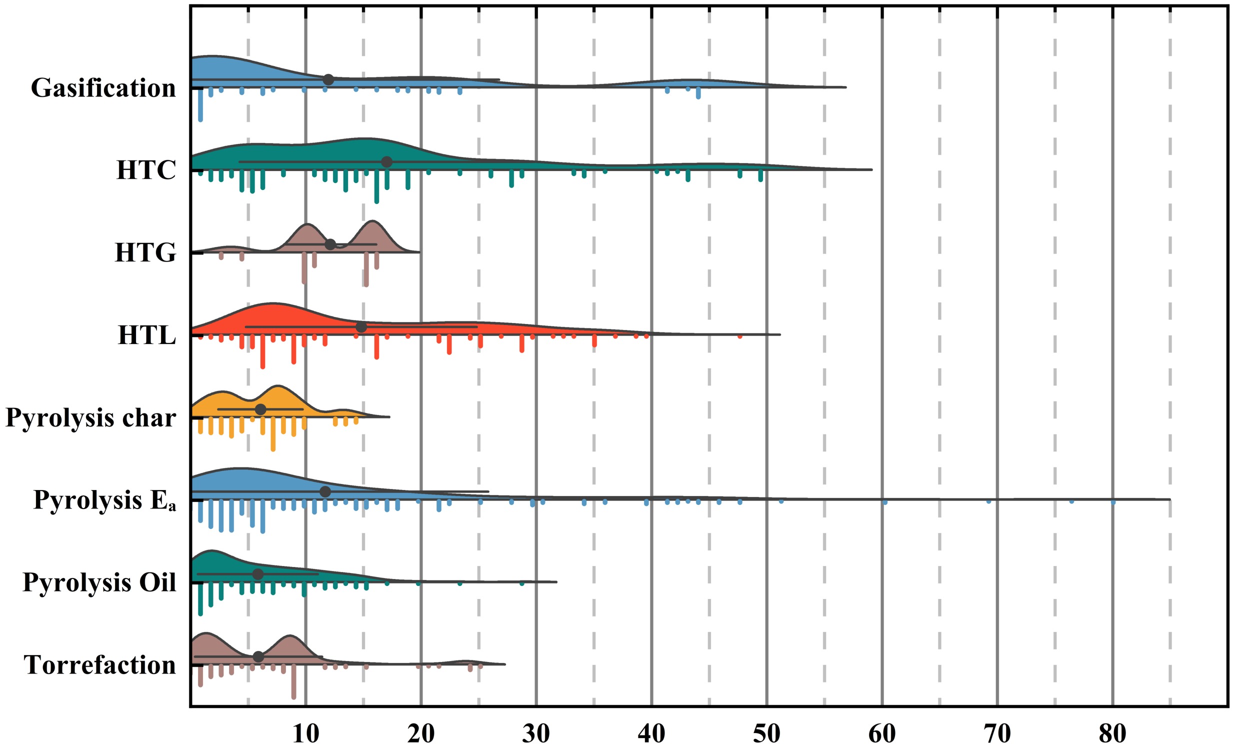


**Supplementary Figure 2. The ash content in the feedstock in the database for each model.**

## Supplementary Note 3

### Feedstock's application as chemicals

The van Krevelen diagrams constructed in this study only provide product information such as its H/C, O/C, yield, etc. This information is useful when considering the product as a fuel but more analysis needs to be conducted when evaluating the potential of using a thermal treatment technique to produce chemicals from biodegradable waste. Again, taking HTC of biodegradable wastes as an example, different reactions lead to the production of different chemical compounds depending on the biomass source and the process conditions. Valuable products such as organic acids, furfurals, phenols, aldehydes, etc., can be produced from the different feedstocks[7].

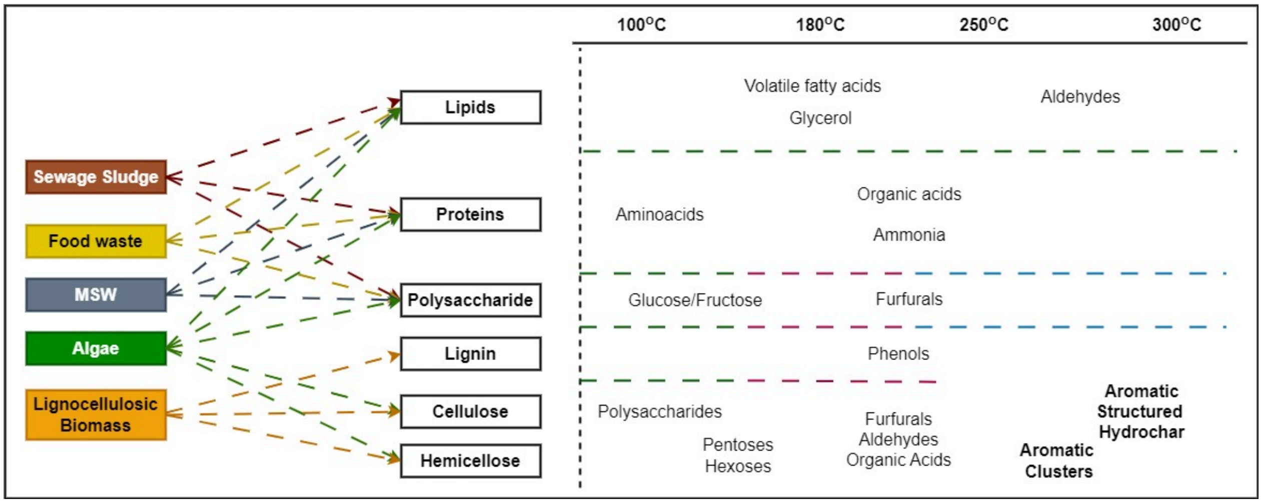


**Supplementary Figure 3. HTC valuable products from different type of feedstocks.** Based on studies reported by Kruse *et al.*[8], Shen[9], Fang et al.[10], Kang et al.[11], Zhuang *et al.*[12] and Usman et al.[7] (MSW: municipal solid waste)

**Supplementary Figure 3** shows the different valuable chemical compounds that can be potentially produced during the HTC of sewage sludge, food waste, municipal solid waste (MSW), algae, and lignocellulosic biomass. These products are strongly related to the process temperature, reaction time and

feedstock's contents of lipids, proteins, polysaccharides, lignin, cellulose and hemicellulose. For instance, biomass with high cellulose, hemicellulose, and polysaccharide composition might produce furfurals (throughout dehydration route) at HTC process temperatures higher that 200°C[13]. Furfurals are considered one of the major platform chemicals that are used in the polymer industry to produce plastics and adhesives, as well as for the production of inks, fertilizers and flavoring compounds. In addition, can be used as a solvent to produce tetrahydrofuran (THF) and levulinic acid (LA)[14]. Sewage sludge due its particular mixing contents of polysaccharides, lipids, and proteins, can promote the formation of organic acids such as amino acids, volatile fatty acids, and nitrogenous compounds such as ammonia[15]. Volatile fatty acids are valuable in the food industry and ammonia can be used as a hydrogen carrier or either as a precursor for the production of fertilizers.

## Supplementary Note 4

### Dataset processing approaches

The Pearson correlation coefficient provides a statistical metric to evaluate the strength and orientation of a linear association between two continuous variables. Values of this coefficient can span from -1 to 1. A coefficient of -1 signifies a perfect inverse linear relationship, 1 denotes a perfect direct linear relationship, and 0 suggests an absence of any linear correlation.

Random Forest Regression (RFR) is a sophisticated machine learning technique that builds multiple decision trees during its training phase. For regression problems, it delivers the mean prediction of these individual trees. As an ensemble learning approach, RFR leverages the predictions from each tree, averaging them to enhance the model's precision and mitigate overfitting. The algorithm's methodology involves the random selection of data subsets and features for every tree, instilling diversity within the forest. This inherent randomness, when amalgamated with the pooling of predictions, equips RFR with resilience against data anomalies and the proficiency to discern intricate non-linear patterns within the dataset.

## Supplementary Note 5

### Using van Krevelen diagrams to evaluate the feedstock-reaction relationships

The methodology implemented in this study can not only create diagrams to reflect the feedstock-product relationship of thermal treatments, but also yield diagrams to indicate the feedstock-reaction relationship. In a previous work, we reported a ML model to predict the model-free pyrolytic activation energy[16]. Based on this model, we followed the methodology used in the current study and created a pyrolytic activation energy diagram as shown in Supplementary Figure 4.

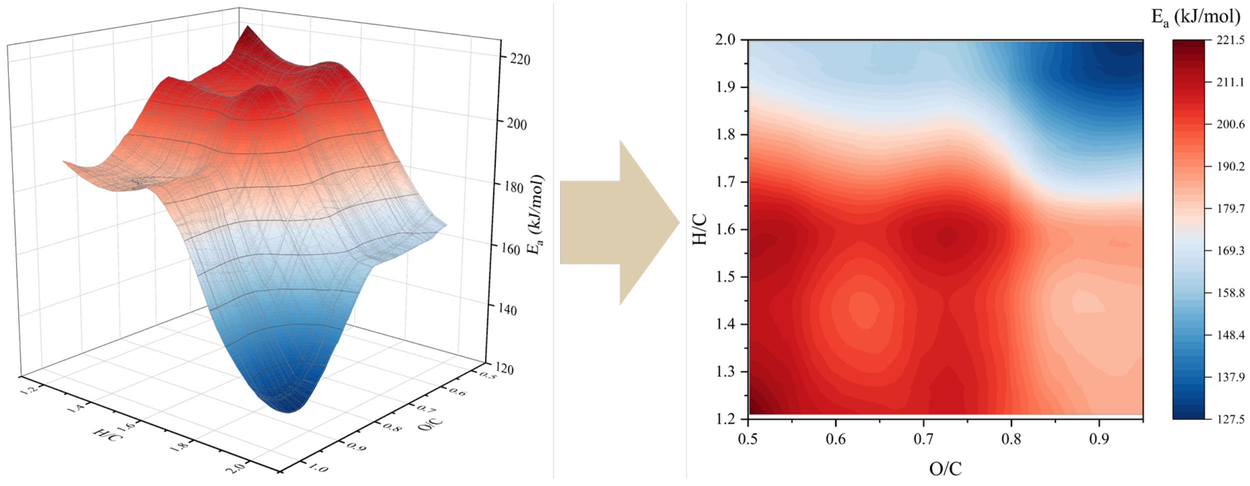


**Supplementary Figure 4. Diagram of activation energy of lignocellulose pyrolysis.** Based on a previous work[16].

The diagram indicates that the pyrolytic activation energy is negatively related to the H/C and O/C values of feedstock. The higher the carbonization degree of the feedstock is, the lower the volatile matter content of the feedstock, and hence, the higher activation energy is required for further thermal decomposition. As a result, it is evident that the methodology used in this study can create van Krevelen diagrams for a better understanding and expression through visualization of reaction mechanisms such as kinetics.

### Smoothing parameters

The smoothing was performed via 3D Smoother App in Origin 2021b. The setup is shown in **Supplementary Figure 5**.

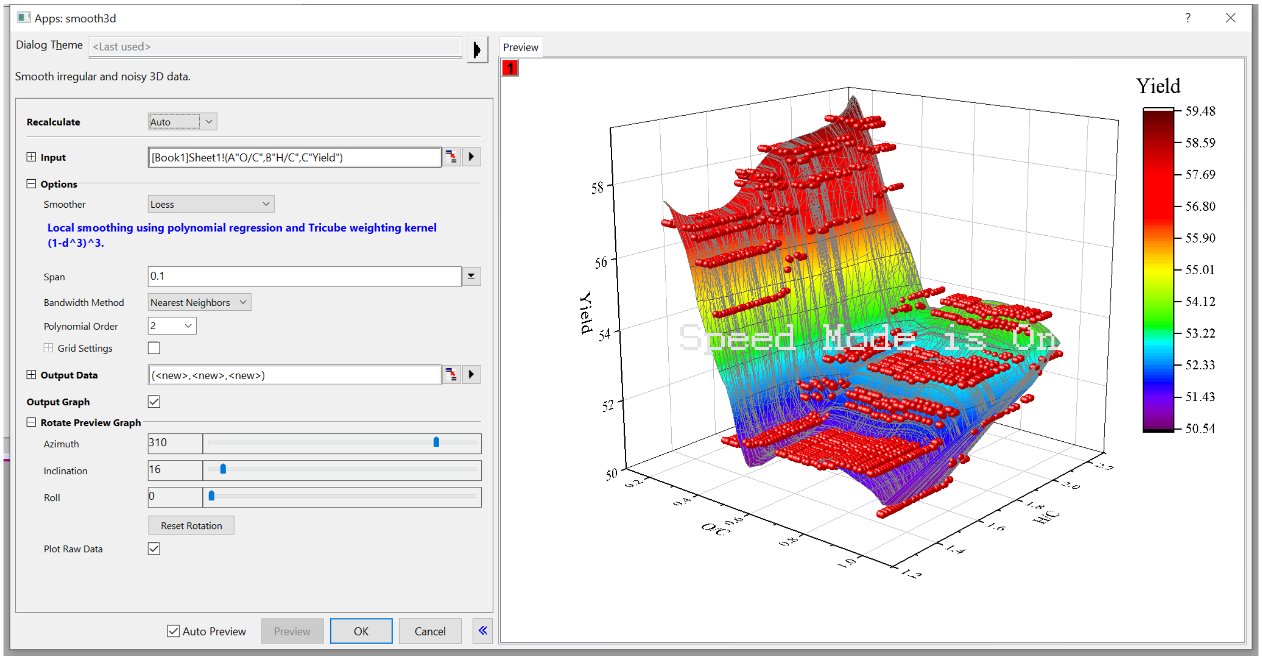


**Supplementary Figure 5. Smoothing setup in Origin 2021b.** Example of producing HTC C diagram. The red spots are the raw data before smoothing.

## Supplementary Note 6

### Reliability analysis of the constructed diagrams

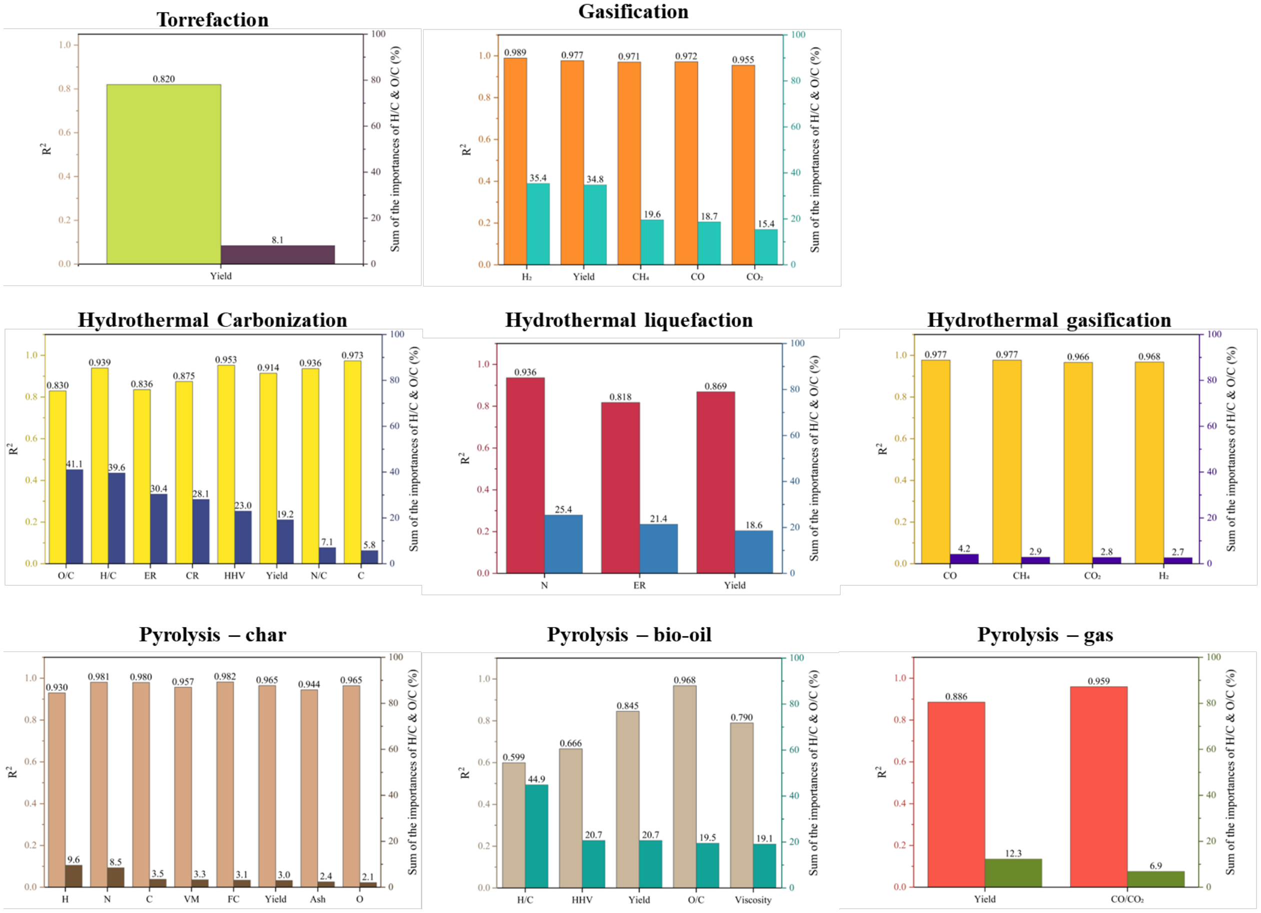


**Supplementary Figure 6 | Determination coefficient $R^2$, as well as the sum of the parameter importance percentages of H/C and O/C of each ML model.** The two variables are used to evaluate the reliability of each yielded van Krevelen diagram.

The reliabilities of the yielded diagrams need to be analyzed to determine the extent to which they can be relied upon. The most important parameter for assessing reliability is the testing accuracies of the ML models, as shown in Supplementary Figure 6. All of the models have $R^2$ values higher than 0.75, except

for the pyrolysis bio-oil models of H/C (0.599) and HHV (0.666). Hence, most of the created models can predict the output variable to a certain degree of accuracy.

Another critical parameter we consider is the weights of the importance of the feedstock's H/C and O/C in relation to the total importance of all input variables. The higher the importance of the feedstock's H/C and O/C, the greater impact these variables have on the output, and therefore, the higher reliability of the diagram. The sums of the importance percentages of feedstock's H/C and O/C in each model are also presented in Supplementary Figure 6. Some models show a very low weight for the sums of H/C and O/C, such as all of the pyrolysis char models with a sum lower than 10%. Although the input importance can only reflect the influence of an input variable on the output in a specific ML model, it can still indicate the importance of the input parameter to some extent in the real situation. For example, the importance percentage of the input variable feedstock's N in the pyrolysis char-N model is 76.8% (Supplementary Figure 15), while the sum of feedstock's O/C and H/C is only 8.5%. It is reasonable to assume that the N content of the char is mainly determined by the initial N content of the feedstocks, while the O/C and H/C can only influence the N content by affecting the relative H and O contents. Based on these, we believe the very low sums of the importance percentages of O/C and H/C inputs in the pyrolysis char models contribute to the unusual patterns observed in the pyrolysis char diagrams. We also do not recommend relying on some of the other diagrams that show reasonable trends but have a low value for this sum.

## Supplementary Note 7

### Kernel density plots of training data

The ML models are constructed using reported experimental data. In these experiments, researchers typically investigate the thermochemical conversion performances of a single feedstock by applying different process parameters. This results in many datasets having the same H/C and O/C input values, but with varying values related to process parameters. Consequently, a direct plot of the training dataset in the van Krevelen diagram does not accurately reflect the real distribution of the dataset, as this diagram only uses H/C and O/C as axes. The Kernel Density Plot, a non-parametric method used to estimate the probability density function of a continuous random variable, is employed in this study to overcome this limitation. It can be used to create a smooth curve from a given set of data.

Torrefaction

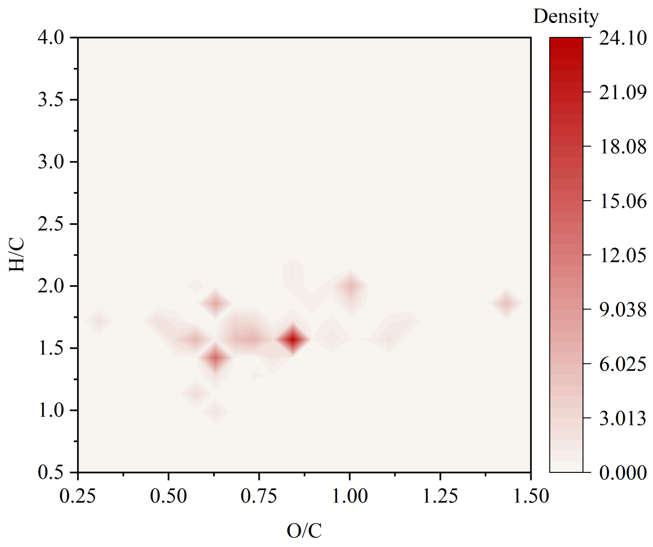


**Supplementary Figure 7. Kernel density plot of training data for torrefaction yield diagram.** Based on study reported by Onsree et al.[17]

Hydrothermal processes

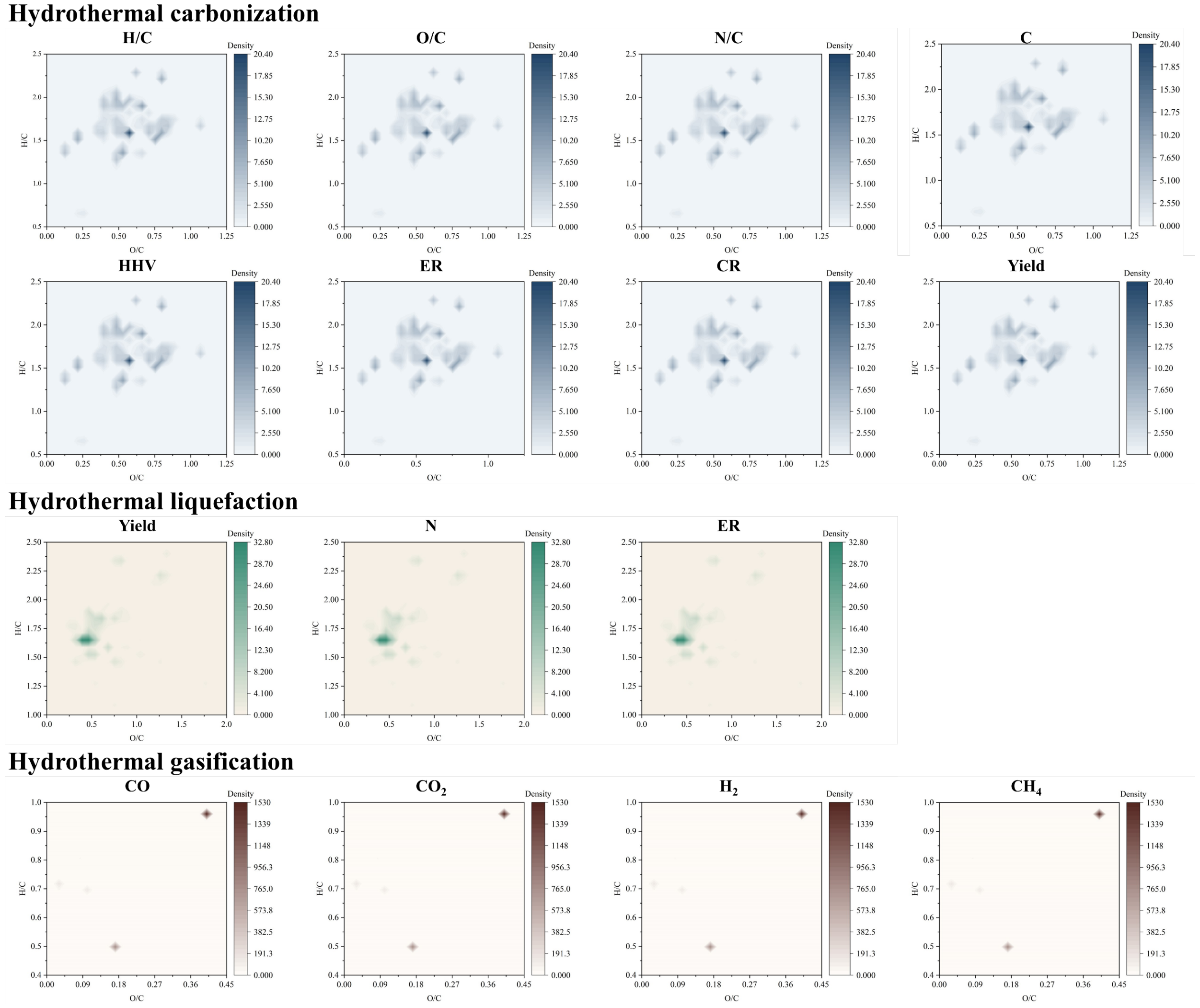


**Supplementary Figure 8. Kernel density plot of training data for HTC, hydrothermal liquefaction (HTL), and hydrothermal gasification (HTG).** Based on studies reported by Li et al.[18,19] and Liu et al.[20]

Pyrolysis

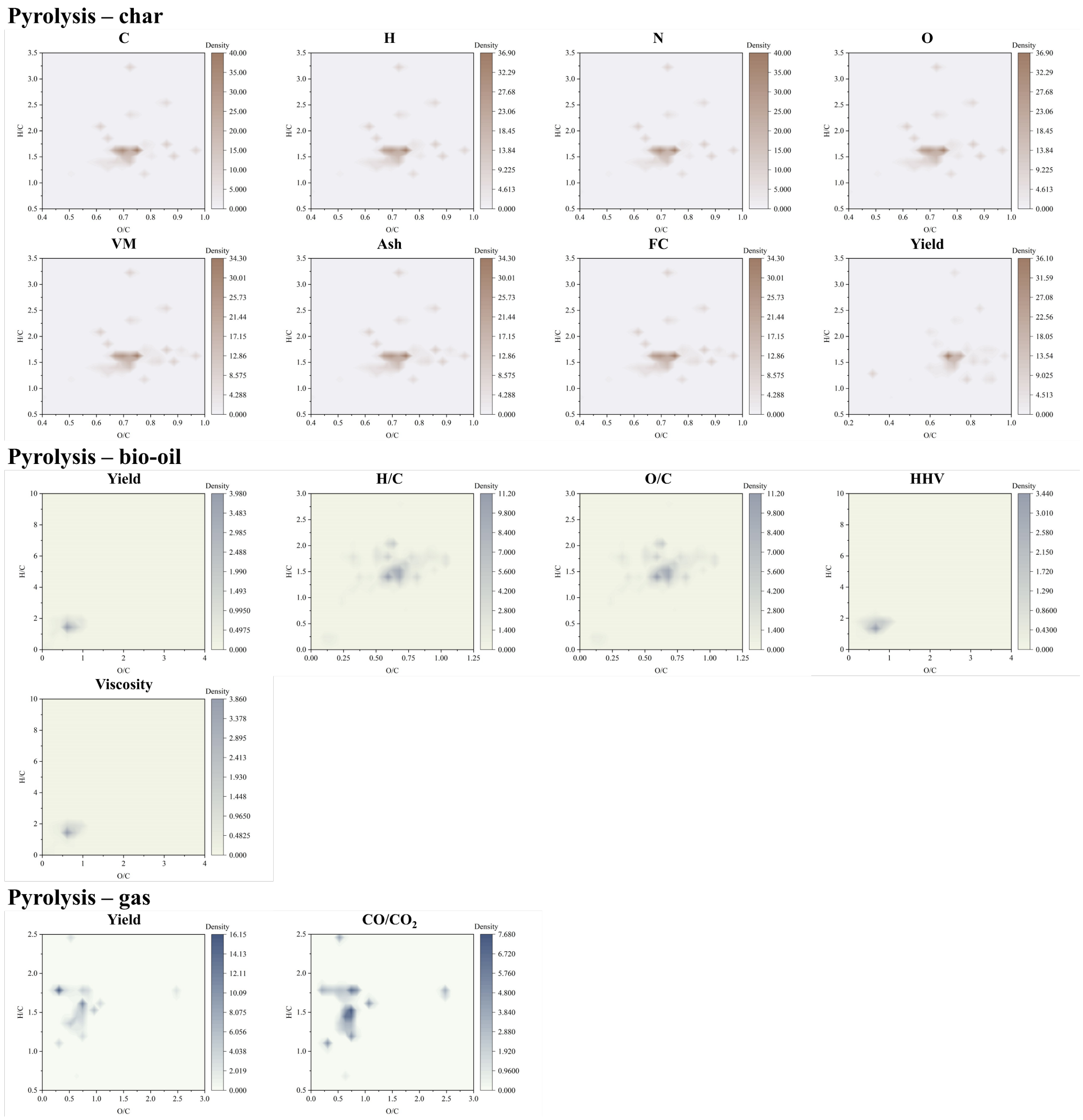


**Supplementary Figure 9. Kernel density plot of training data for pyrolysis.** Based on studies reported by Li et al.[21], Zhang et al.[22], and Tang et al.[23]

Gasification

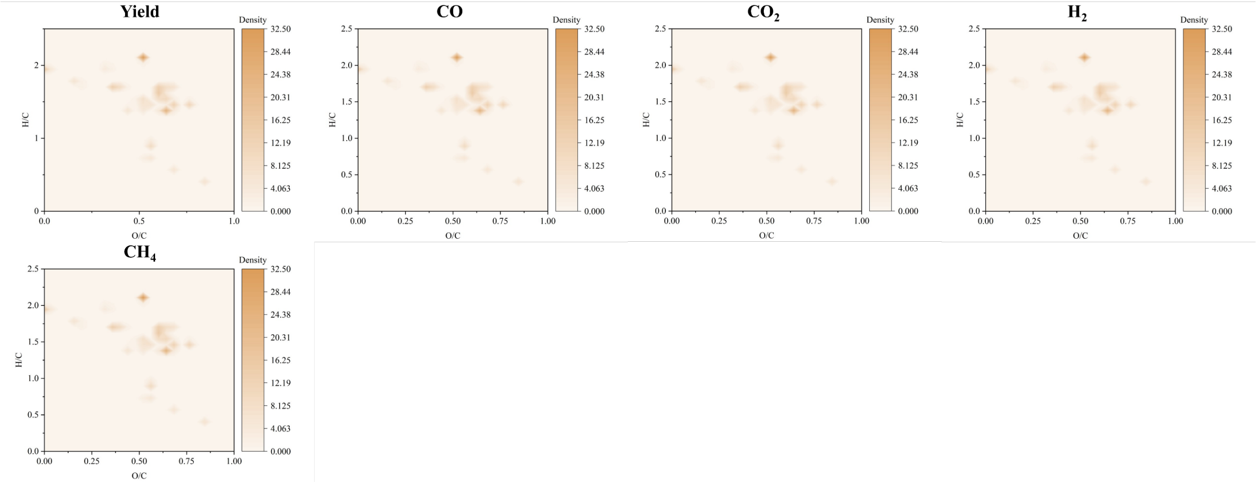


**Supplementary Figure 10. Kernel density plot of training data for gasification.** Based on studies reported by Serrano et al.[24]

## Supplementary Note 8

### Fitting results and the interpretation of models

Random Forest Regression is an advanced ensemble learning method that constructs multiple decision trees during its training phase. For regression tasks, it outputs the mean prediction of these individual trees. A salient feature of the Random Forest is its intrinsic capacity to rank the significance of features utilized in predictions. This not only enhances model precision but also sheds light on the intrinsic patterns and associations within the dataset.

The determination of feature importance in a Random Forest is anchored on the average decrement in node impurity, predominantly gauged using the Mean Squared Error (MSE) in regression contexts. Fundamentally, the greater the reduction in impurity a feature facilitates, the more pivotal it becomes. Throughout each tree's formation in the forest, every feature split in every tree is recorded. A feature's importance is ascertained by assessing the average reduction in MSE brought about by tree nodes that utilize this feature for splitting. Subsequently, these values are averaged across all trees, culminating in a hierarchy of feature significance.

Grasping feature importance is imperative for model interpretability. It empowers researchers and professionals to discern the most influential predictors in the prediction process. Such understanding not only streamlines the model by emphasizing pivotal features but also bestows sector-specific insights, a treasure trove in disciplines such as medicine, finance, and ecology. Furthermore, by pinpointing and discarding less consequential features, one can sculpt a more streamlined model, potentially bolstering its generalization capabilities and curtailing overfitting.

Torrefaction

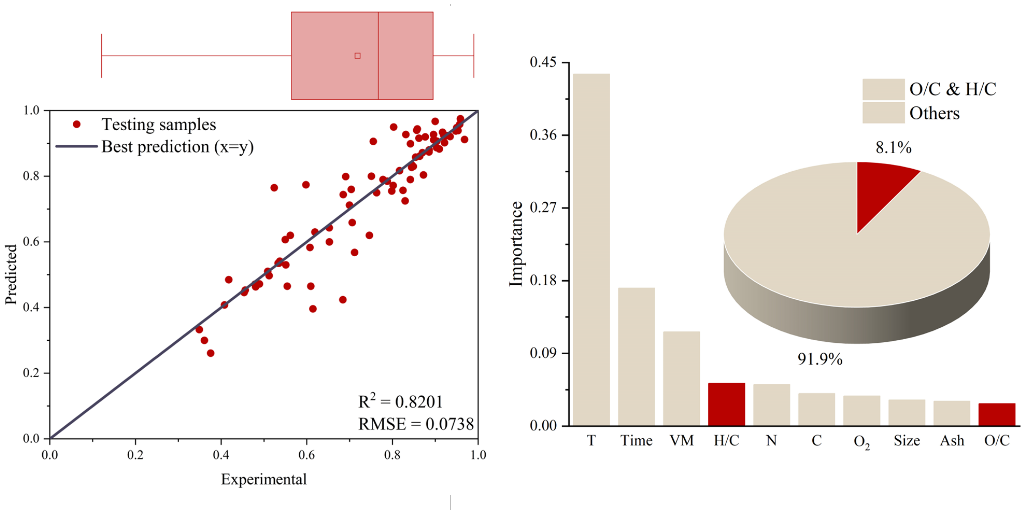


**Supplementary Figure 11. Fitting result and importances of H/C and O/C of torrefaction model.**

Hydrothermal carbonization

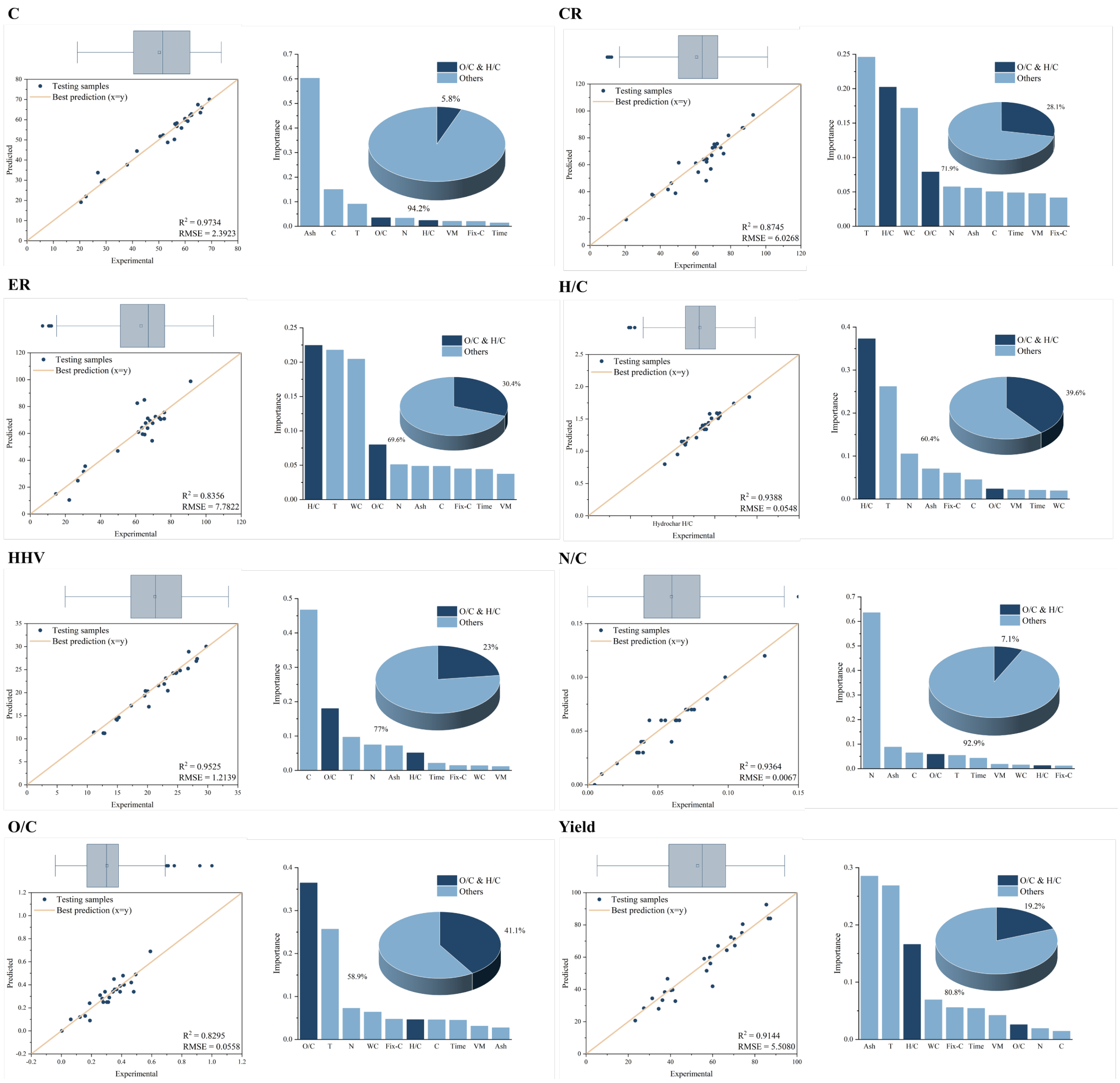


**Supplementary Figure 12. Fitting results and importances of H/C and O/C of HTC models.**

Hydrothermal liquefaction

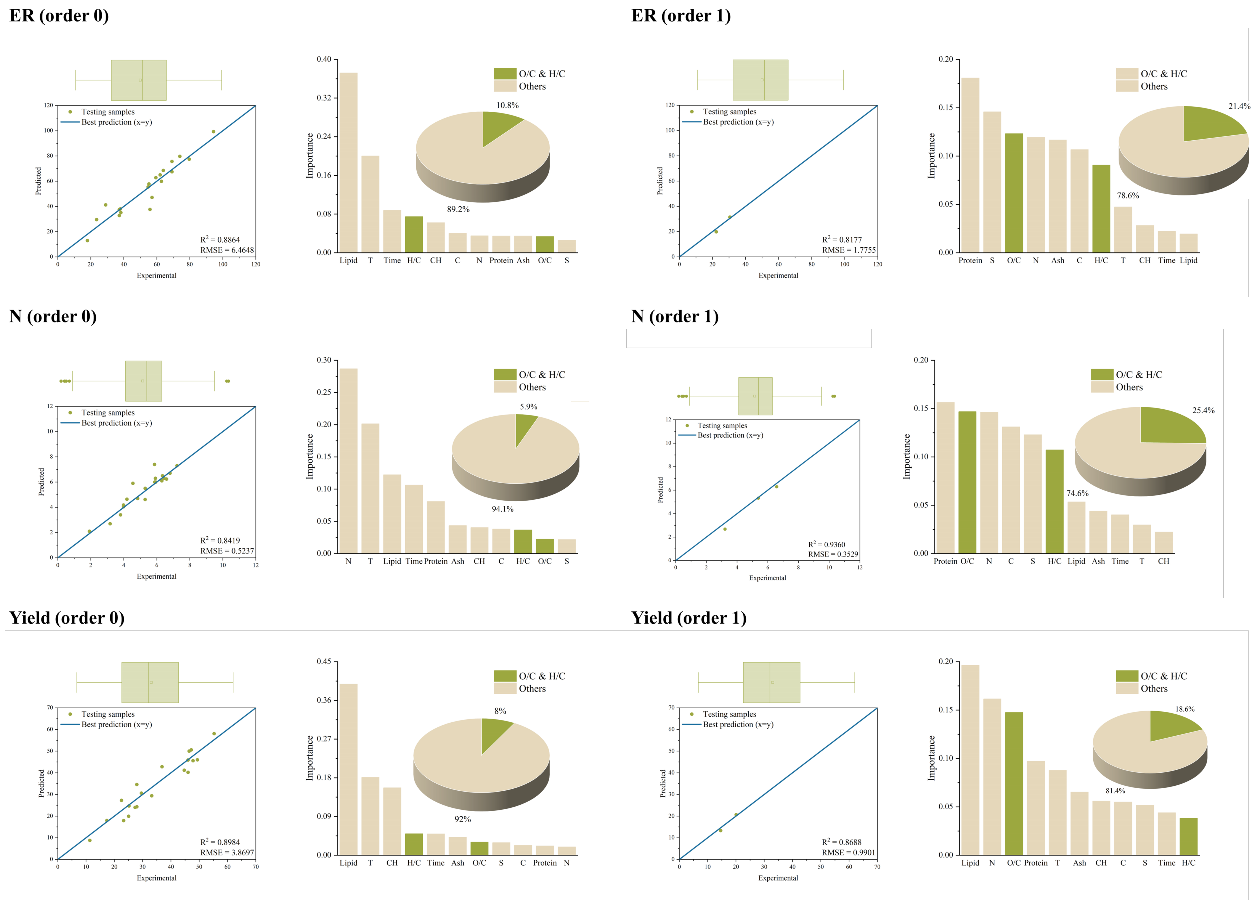


**Supplementary Figure 13. Fitting results and importances of H/C and O/C of HTL models.**

Hydrothermal gasification

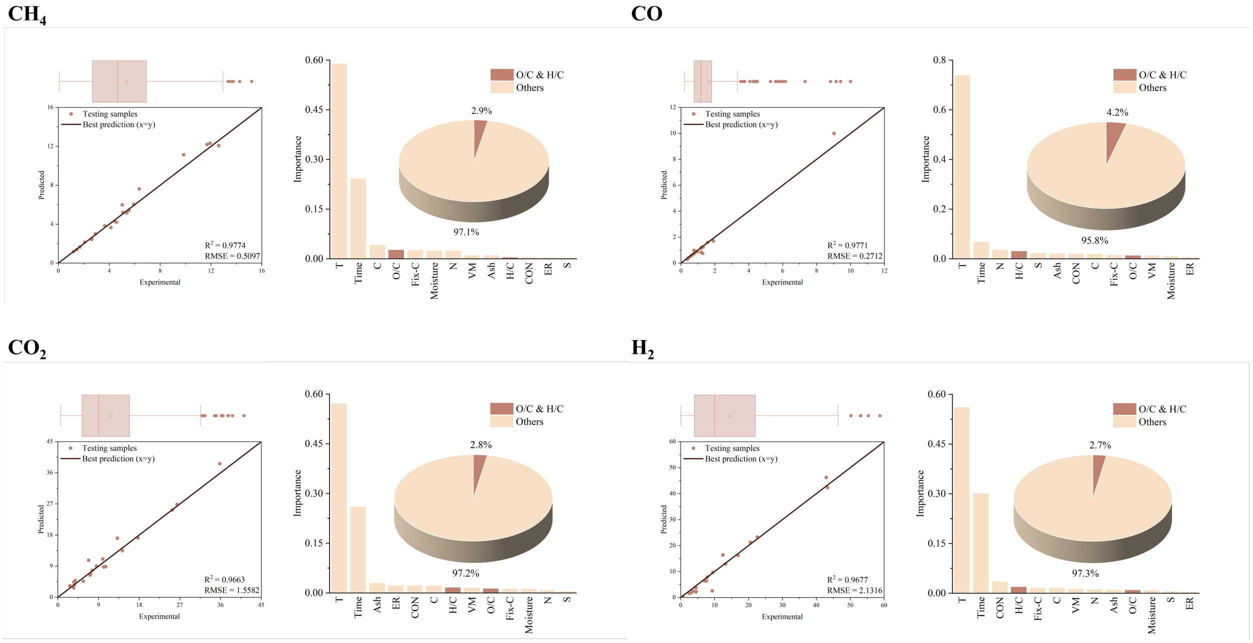


**Supplementary Figure 14. Fitting results and importances of H/C and O/C of HTG models.**

## Pyrolysis – char

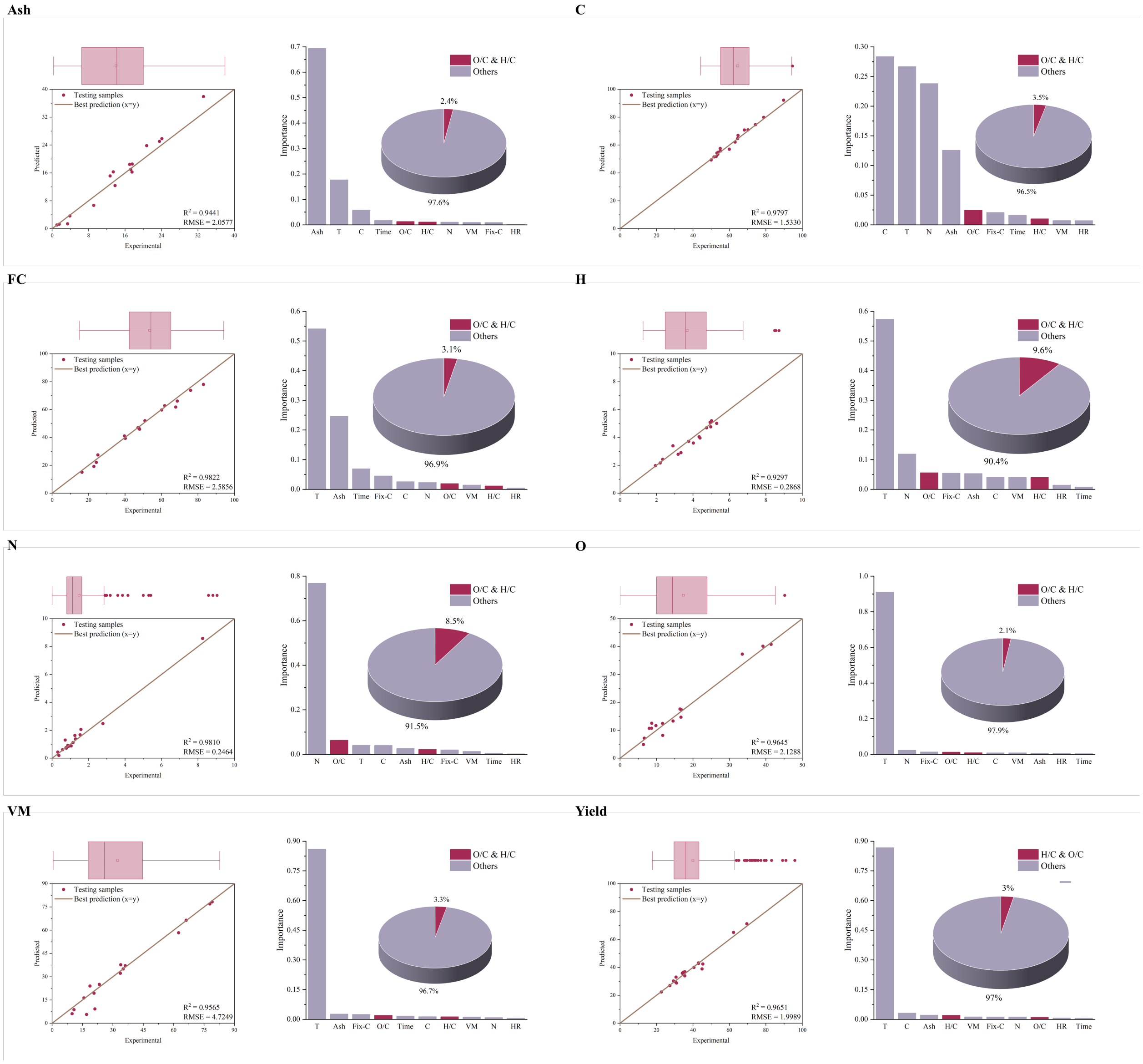


**Supplementary Figure 15. Fitting results and importances of H/C and O/C of pyrolysis – char models.**

Pyrolysis – bio-oil

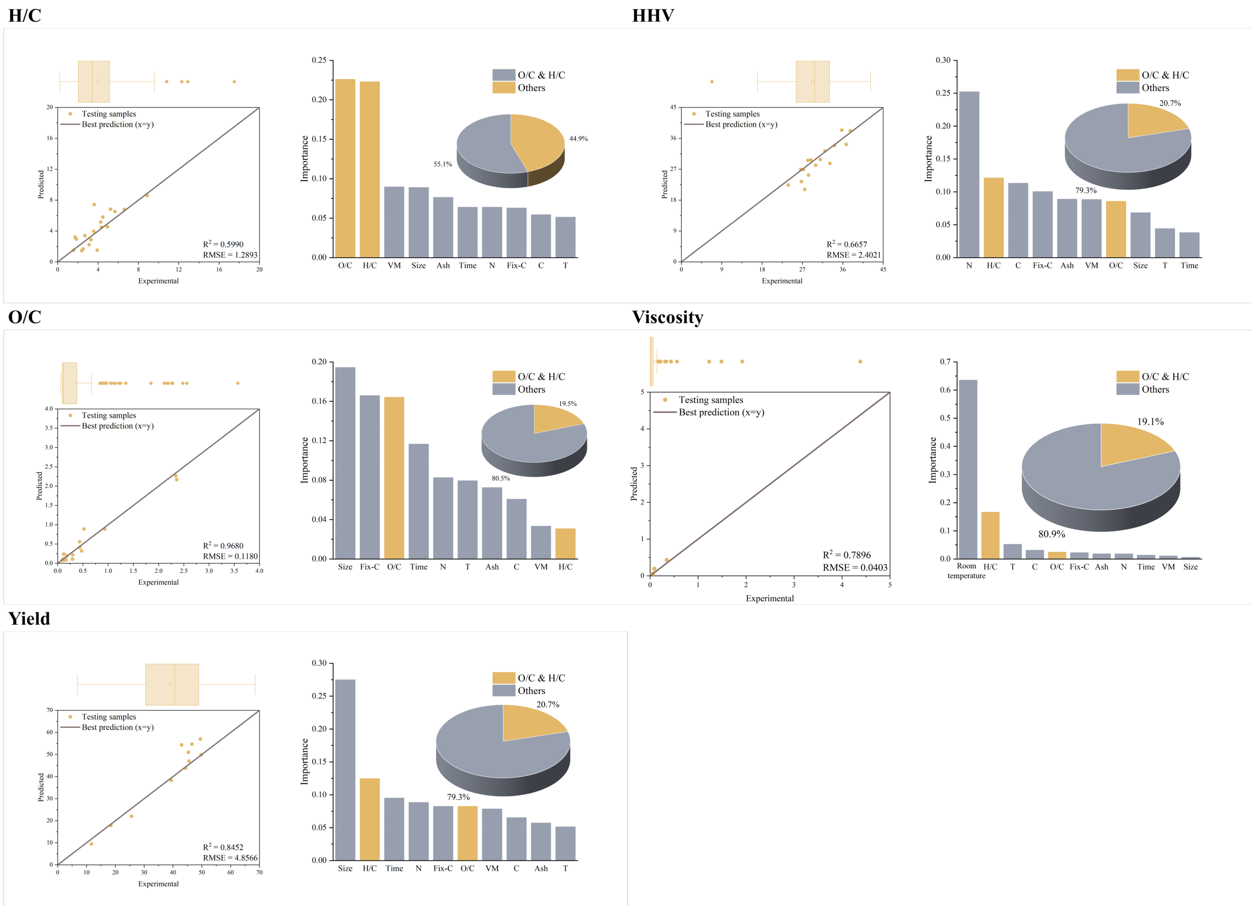


**Supplementary Figure 16. Fitting results and importances of H/C and O/C of pyrolysis – bio-oil models.**

Pyrolysis – gas

**$CO/CO_2$**

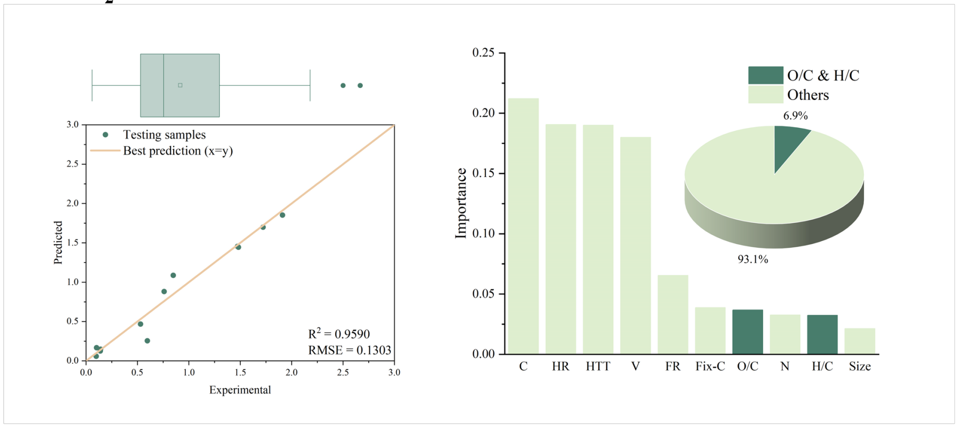


**Yield**

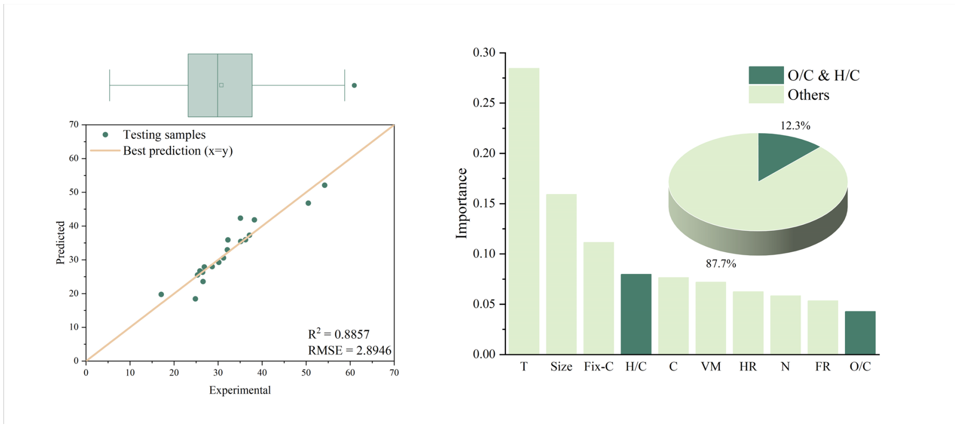


**Supplementary Figure 17. Fitting results and importances of H/C and O/C of pyrolysis – gas models.**

Gasification

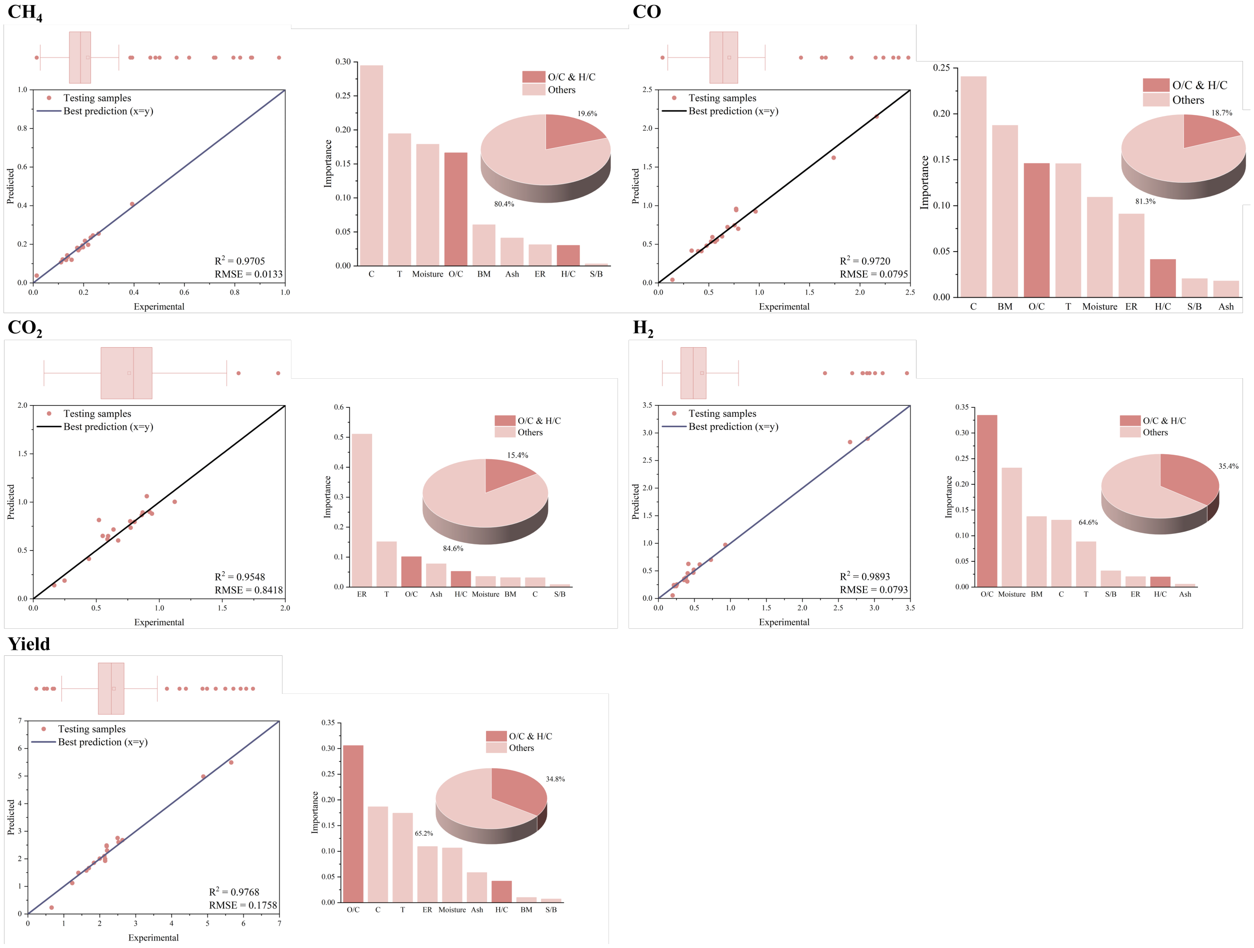


**Supplementary Figure 18. Fitting results and importances of H/C and O/C of gasification models.**

## Supplementary References


1 Sharma, H. B., Panigrahi, S. & Dubey, B. K. Food waste hydrothermal carbonization: Study on the effects of reaction severities, pelletization and framework development using approaches of the circular economy. *Bioresource Technology* **333**, 125187 (2021).

2 Wang, L. *et al.* Hydrothermal treatment of food waste for bio-fertilizer production: Formation and regulation of humus substances in hydrochar. *Science of The Total Environment* **838**, 155900 (2022).

3 Djandja, O. S., Yin, L.-X., Wang, Z.-C. & Duan, P.-G. From wastewater treatment to resources recovery through hydrothermal treatments of municipal sewage sludge: A critical review. *Process Safety and Environmental Protection* **151**, 101-127 (2021).

4 Wang, T. *et al.* Co-hydrothermal carbonization of food waste-woody biomass blend towards biofuel pellets production. *Bioresource technology* **267**, 371-377 (2018).

5 Yildiz, G. *et al.* Effect of biomass ash in catalytic fast pyrolysis of pine wood. *Applied Catalysis B: Environmental* **168**, 203-211 (2015).

6 Shahbaz, M., Inayat, A., Patrick, D. O. & Ammar, M. The influence of catalysts in biomass steam gasification and catalytic potential of coal bottom ash in biomass steam gasification: a review. *Renewable and Sustainable Energy Reviews* **73**, 468-476 (2017).

7 Usman, M. *et al.* Characterization and utilization of aqueous products from hydrothermal conversion of biomass for bio-oil and hydro-char production: a review. *Green chemistry* **21**, 1553-1572 (2019).

8 Kruse, A., Funke, A. & Titirici, M.-M. Hydrothermal conversion of biomass to fuels and energetic materials. *Current opinion in chemical biology* **17**, 515-521 (2013).

9 Shen, Y. A review on hydrothermal carbonization of biomass and plastic wastes to energy products. *Biomass and Bioenergy* **134**, 105479 (2020).

10 Kang, S., Li, X., Fan, J. & Chang, J. Characterization of hydrochars produced by hydrothermal carbonization of lignin, cellulose, D-xylose, and wood meal. *Industrial & engineering chemistry research* **51**, 9023-9031 (2012).

11 Fang, Z. *et al.* Reaction chemistry and phase behavior of lignin in high-temperature and supercritical water. *Bioresource Technology* **99**, 3424-3430 (2008).

12 Zhuang, X. *et al.* Insights into the evolution of chemical structures in lignocellulose and non-lignocellulose biowastes during hydrothermal carbonization (HTC). *Fuel* **236**, 960-974 (2019).

13 Remón, J., Santomauro, F., Chuck, C. J., Matharu, A. S. & Clark, J. H. Production of fermentable species by microwave-assisted hydrothermal treatment of biomass carbohydrates: reactivity and fermentability assessments. *Green Chemistry* **20**, 4507-4520 (2018).

14 Mathew, A. K., Abraham, A., Mallapureddy, K. K. & Sukumaran, R. K. in *Waste biorefinery* 267-297 (Elsevier, 2018).

15 Esquinas-Requena, J. L. *et al.* La anemia aumenta el riesgo de mortalidad debido a fragilidad y discapacidad en mayores: Estudio FRADEA. *Atencion primaria* **52**, 452-461 (2020).

16 Wang, S. *et al.* A machine learning model to predict the pyrolytic kinetics of different types of feedstocks. *Energy Conversion and Management* **260**, 115613 (2022).

17 Onsree, T. & Tippayawong, N. Machine learning application to predict yields of solid products from biomass torrefaction. *Renewable Energy* **167**, 425-432 (2021).

18 Li, J. *et al.* Multi-task prediction and optimization of hydrochar properties from high-moisture municipal solid waste: Application of machine learning on waste-to-resource. *Journal of Cleaner Production* **278**, 123928 (2021).

19 Li, J. *et al.* Machine learning aided bio-oil production with high energy recovery and low nitrogen content from hydrothermal liquefaction of biomass with experiment verification. *Chemical Engineering Journal* **425**, 130649 (2021).

20 Liu, S. *et al.* Predicting gas production by supercritical water gasification of coal using machine learning. *Fuel* **329**, 125478 (2022).

21 Li, Y., Gupta, R. & You, S. Machine learning assisted prediction of biochar yield and composition via pyrolysis of biomass. *Bioresource Technology* **359**, 127511 (2022).

22 Zhang, T. *et al.* Machine learning prediction of bio-oil characteristics quantitatively relating to biomass compositions and pyrolysis conditions. *Fuel* **312**, 122812 (2022).

23 Tang, Q. *et al.* Machine learning prediction of pyrolytic gas yield and compositions with feature reduction methods: Effects of pyrolysis conditions and biomass characteristics. *Bioresource technology* **339**, 125581 (2021).

24 Serrano, D., Golpour, I. & Sánchez-Delgado, S. Predicting the effect of bed materials in bubbling fluidized bed gasification using artificial neural networks (ANNs) modeling approach. *Fuel* **266**, 117021 (2020).
