# Supplementary material for: Van Krevelen diagrams based on machine learning visualize feedstock-product relationships in thermal conversion processes
Source: Commun Chem. 2023 Dec 13;6:273. doi: 10.1038/s42004-023-01077-z (PMC10716171; doi:10.1038/s42004-023-01077-z)
Supplement: Supplementary file 3 — Description of Additional Supplementary Files [file 42004_2023_1077_MOESM3_ESM.pdf]

Description of Additional Supplementary Files

File name: Supplementary Data 1

Description: Dataset for the yields of torrefied biomass with their corresponding features.
